# Adaptive-like features of the γδ TCR couple chronic BTNL recognition to NK-like tissue immunity

**DOI:** 10.64898/2026.09.17.751481

**Published:** 2026-09-24

**Authors:** Benjamin D McDonald, Hope D Anderson, Toufic Mayassi, Chhon Ling Sok, Veronica Locher, Magdalena Justyniarska, Megan E Borregard, Caroline Kaiser, Kristin Ladell, James E McLaren, David A Price, Narutoshi Hibino, Andrew S Koh, Jamie Rossjohn, Benjamin S Gully, Samantha J Riesenfeld, Bana Jabri

## Abstract

Intestinal Vγ4 γδ intraepithelial lymphocytes (IELs) persistently bind the constitutively expressed epithelial ligand BTNL3/8 through germline-encoded T cell receptor (TCR) determinants. While they resemble innate-like T cells such as NKT cells, unlike these populations, Vγ4 IELs encounter ligand only after thymic development. Moreover, unlike conventional αβ T cells, Vγ4 IELs sustain persistent physiological ligand engagement without becoming exhausted. Here, using biophysical, functional, and multimodal single-cell approaches, we show how Vγ4 IELs address this challenge. While germline-encoded TCR regions broadly mediate BTNL3 recognition, productive activation requires additional non-germline TCR features that license responsiveness to BTNL3/8 and enable local selection in the gut. Rather than driving exhaustion, BTNL3/8 reactivity directly promotes expression of an NK-like program marked by adaptor molecules that license innate-like signaling in healthy tissue. These findings reveal how combined innate and adaptive features of the γδTCR enable durable tissue specialization under conditions of persistent physiological ligand engagement.

## Full Text Availability

The license terms selected by the author(s) for this preprint version do not permit archiving in PMC. The full text is available from the preprint server.

